# A machine learning model for predicting the ballistic impact resistance of unidirectional fiber-reinforced composite plate

**DOI:** 10.1038/s41598-021-85963-3

**Published:** 2021-03-22

**Authors:** X. D. Lei, X. Q. Wu, Z. Zhang, K. L. Xiao, Y. W. Wang, C. G. Huang

**Affiliations:** 1 Key Laboratory for Mechanics in Fluid Solid Coupling Systems, Institute of Mechanics, Chinese Academy of Sciences, Beijing, 100190 China; 2 School of Engineering Science, University of Chinese Academy of Sciences, Beijing, 100049 China; 3 Hefei Institutes of Physical Science, Chinese Academy of Sciences, Heifei, 230031 China

**Keywords:** Structural materials, Mechanical engineering

## Abstract

It has been a vital issue to ensure both the accuracy and efficiency of computational models for analyzing the ballistic impact response of fiber-reinforced composite plates (FRCP). In this paper, a machine learning (ML) model is established in an effort to bridge the ballistic impact protective performance and the characteristics of microstructure for unidirectional FRCP (UD-FRCP), where the microstructure of the UD-FRCP is characterized by the two-point correlation function. The results showed that the ML model, after trained by 175 cases, could reasonably predict the ballistic impact energy absorption of the UD-FRCP with a maximum error of 13%, indicating that the model can ensure both computational accuracy and efficiency. Besides, the model’s critical parameter sensitivities are investigated, and three typical ML algorithms are analyzed, showing that the gradient boosting regression algorithm has the highest accuracy among these algorithms for the ballistic impact problem of UD-FRCP. The study proposes an effective solution for the traditional difficulty of the ballistic impact simulation of composites with both high efficiency and accuracy.

## Introduction

Fiber-reinforced composite plate (FRCP) has been widely used in engineering involving aircraft structure, civil construction, ocean engineering, etc., due to its high specific strength and modulus^1–5^. In addition, the numerous energy dissipation channels including tension and failure of fibers, friction between fibers and matrix, and compression failure of matrix render FRCP excellent impact energy dissipation capacity^6–8^, making it a preferred material in impact protective structures such as bulletproof vests and vehicles.

The impact resistance of FRCP is dependent on the dynamic behavior of fibers and matrix, and it is affected significantly by the microstructure. Generally, there is a consensus that unidirectional FRCP (UD-FRCP) can provide higher impact resistance than other structural topologies^9^ for the bulletproof composite. In addition to ballistic impact tests, numerical simulation has been proven to be an effective method for predicting the impact resistance of UD-FRCP^10–12^. The simulation method can be classified into two scales, i.e. macro-scale^13^ and micro-scale^14,15^, according to the modeling characteristics. For the macro-scale simulation of FRCP implemented by the finite element analysis (FEA), the transverse isotropic constitutive model is usually used for each layer of UD-FRCP in cooperation with some failure criteria such as Hashin failure^16^ to analyze the ballistic impact responses. For this method, the results such as ballistic limit velocity and damage morphology always agree with the experimental results according to the study by Abdel-Nasser et al.^17^, Zhu et al.^18^, etc. However, it is hard to obtain the evolution of composite microstructures during impact like damage behavior induced by the ballistic impact. To capture the detailed deformation and failure behavior of composites to increase the accuracy of computation, the micro-scale simulations are generally performed, in which fiber bundles, matrix, and architectures are modeled. According to Refs.^19,20^ that are simulated by micro-scale models, the failure criteria of the matrix and the interfacial condition play a dominant role in the phenomenological failure criteria of the composite. The failure behaviors and the damage evolution of the composites are also clearly delineated during penetration. However, it is worth noting that the computation at micro-scale generally takes more than 2000 h CPU-time for one case^21^, which is extremely time-consuming when compared to the macro-scale simulation. As can be seen from the literature mentioned above, the computational efficiency of the macro-scale models is high, whereas the accuracy is generally not satisfied. In contrast, the computational accuracy of the micro-scale models is usually satisfactory, but the computational cost is too expensive for a model with relatively large dimensions. Therefore, it is crucial to put forward computation models with both high efficiency and accuracy for predicting the ballistic impact resistance of UD-FRCP.

In recent years, with the rapid development of computing science and technology, machine learning (ML) method begin to be widely adopted to predict the mechanical behavior of various materials, providing a promising solution for obtaining a computation model with both high efficiency and accuracy to obtain the ballistic impact resistance of UD-FRCP. The availability of supervised ML and convolutional neural networks have been verified in depicting the relationship between materials, structures, and mechanical properties^22,23^ such as stress–strain curve, modulus of elasticity, and yield strength. The development of ML greatly broadens the research method of mechanics. However, the previous prediction studies by the ML method mainly focus on the quasi-static mechanical properties of composites. Whether the ML method can reasonably predict the ballistic impact behavior of UD-FRCP is still unknown.

In this paper, the ballistic impact resistance of UD-FRCP is studied by a ML method for the first time. The critical parameter sensitivities, various ML algorithms, and the influence of the number of training cases are also investigated. The results show that the ballistic impact resistance of UD-FRCP can be well predicted by the proposed model. The paper is organized as follows. “Model and methodology” gives the details of the ML model. In “Results and discussion”, the prediction results are provided. The sensitivities of parameters are investigated, the effects of various ML algorithms are also compared, and the influence of the number of cases on the prediction error is demonstrated, followed by the discussion and conclusions.

## Model and methodology

**Figure 1:**
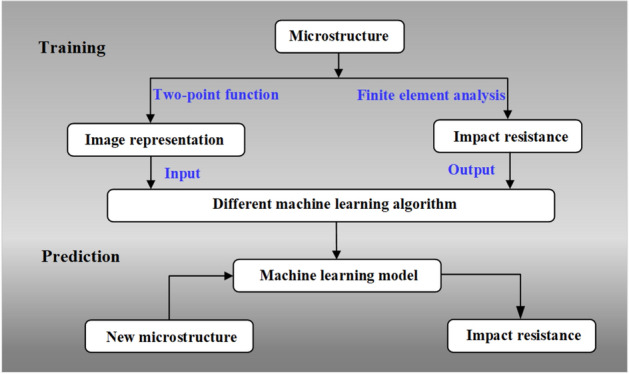
Flow diagram of the analysis method.

The detailed process of the ML model is shown in Fig. 1. Firstly, various microstructures of UD-FRCP are generated and characterized. The ballistic impact database of the UD-FRCP with different microstructure is then built based on massive FEA simulations. After that, three typical ML algorithms are employed to predict the ballistic impact resistance of the UD-FRCP with a given microstructure characteristic, and the sensitivities of the related parameters are investigated. Finally, a ML model with both high efficiency and accuracy is proposed for predicting the ballistic impact resistance of UD-FRCP.

### Numerical model

#### Construction of UD-FRCP

**Figure 2:**
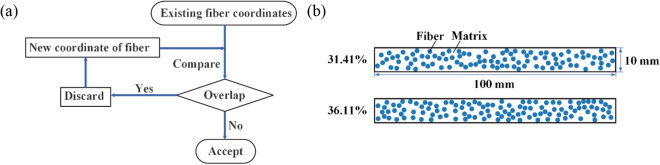
(**a**) Flow chart of microstructure generation. (**b**) Microstructure images of 31.41% and 36.11% volume fractions of fibers by changing the different number of fiber bundles in the cross-section of the UD-FRCP.

We randomly generated 185 UD-FRCP materials with different cross-section characteristics with 18 ~ 48% fiber content. The method of microstructure generation is shown in Fig. 2a. Several microstructures are generated for the same fiber content in order to explore the influence of the fiber distribution characteristics on the impact protective performance of the composite materials. The typically generated microstructures are shown in Fig. 2b, where the blue circles represent the fiber bundles with a diameter of 2 mm, and the rest regions are the matrix. The fiber bundle and matrix modeling strategy has been adopted in some micro-scale models^11,24^. In addition, Chocron et al.^11^ has constructed the unidirectional composites model by bundling fibers into strips. The ratio of the width of the fiber bundle (i.e. 0.67 mm) to the projectile diameter (i.e. 2 mm) is about 1/3, which is much larger than that of our present study (2-mm-diameter fiber bundle and 30-mm-diameter projectile, leading to the ratio of 1/15). Therefore, this scheme is feasible in the micro-scale model by adopting the fiber bundle with a diameter of 2 mm. The dimensions of the rectangle cross-section are 100 × 10 mm. The volume fractions of the fibers can be adjusted by changing the number of fiber bundles in the cross-section. With this method, 100 fiber bundles lead to a 31.41% volume fraction, and 115 fiber bundles lead to a 36.11% volume fraction, as shown in Fig. 2b.

#### Micro-scale ballistic impact model

**Figure 3:**
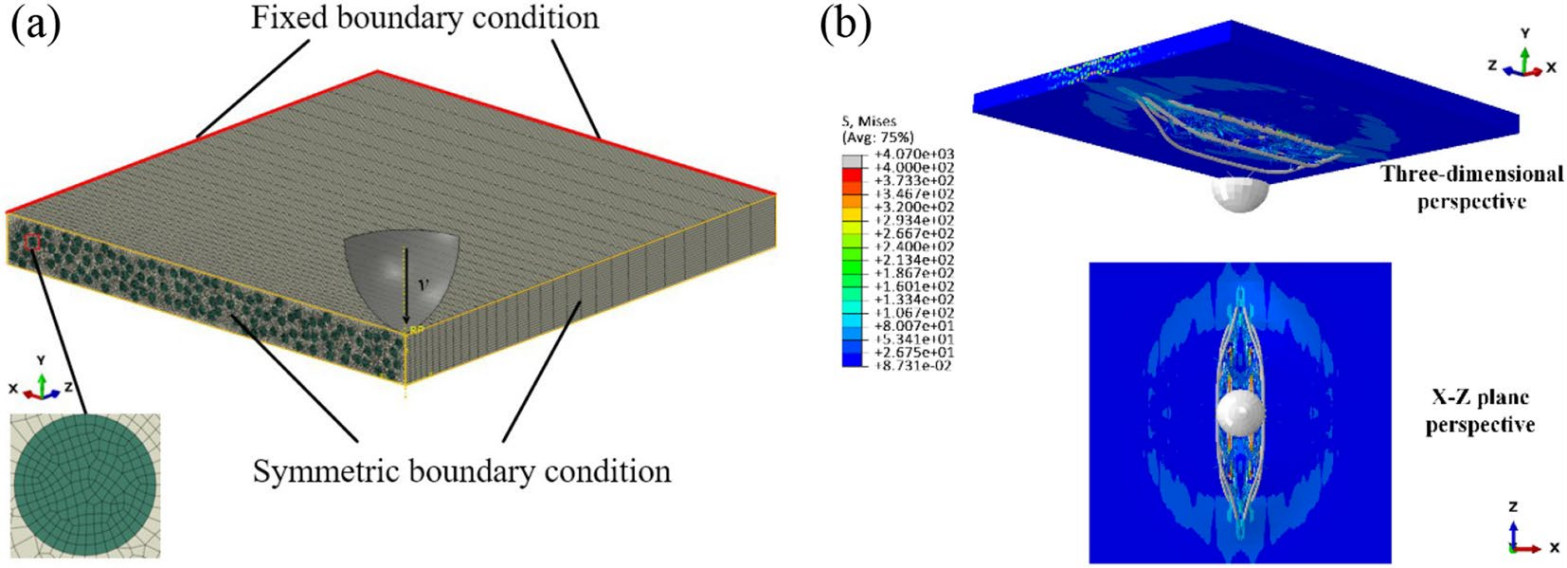
(**a**) The ¼ micro-scale impact model of the UD-FRCP. (**b**) The typical simulation results of the micro-scale model.

The ABAQUS explicit software, which has been widely used in the impact response of composites^25–27^, is employed to analyze the ballistic impact response of the UD-FRCP. As shown in Fig. 3a, a 1/4 model with the dimensions of 100 × 100 × 10 mm is built due to the symmetries of the problem to improve computational efficiency. In addition, since hundreds of impact processes will be simulated to create the database for ML, a 1/8 rigid spherical surface with a mass of 55.8 g and a radius of 15 mm is taken as a projectile to mimic the bullet to decrease the computation cost further. The mesh sizes of the fibers and the matrix are matched, which are both 0.16 mm in the cross-sectional direction. In the extensional direction of the length, the size gradually transforms from 0.5 mm to 6.65 mm in order to reduce the number of meshes and consider the main effects of numerically simulated impact position. The meshes of the fiber bundle and the matrix exceed 620,000 and 640,000 C3D8R elements in the 1/4 model, respectively. The initial impact velocity of the projectile is 300 m/s. The symmetrical boundary conditions are applied to the symmetrical cross-sections of the UD-FRCP and the projectile, and the fixed boundary condition is applied to the peripheries of the UD-FRCP far away from the projectile. The general contact condition is used to model the non-linear contact behavior between the projectile and the UD-FRCP during penetration. Honestly, the ballistic impact models for the UD-FRCP are significantly simplified in an effort to achieve the highest computational efficiency. However, the effects of microstructures of the cross-sections on the impact protective performance of the UD-FRCP can still be revealed. One could expect better results from fine-meshed practical micro-scale models. Nevertheless, it will be quite time-consuming while building the database for ML, which will be performed in the future. Here, we mainly focus on the validation of the ML method for analyzing the impact protective performance of UD-FRCP with various microstructures.

The Tsai-Wu failure criterion^28^ is undertaken in ABAQUS explicit package^29^ to describe the failure behavior of the UD-FRCP,

1 $$ F_{1} \sigma_{11} + F_{2} \sigma_{22} + F_{3} \sigma_{33} + F_{11} \sigma_{11}^{2} + F_{22} \sigma_{22}^{2} + F_{33} \sigma_{33}^{2} + F_{44} \sigma_{23}^{2} + F_{55} \sigma_{13}^{2} + F_{66} \sigma_{12}^{2} + F_{12} \sigma_{11} \sigma_{22} + F_{13} \sigma_{11} \sigma_{33} + F_{23} \sigma_{22} \sigma_{33} \ge 1. $$

The failure parameters are defined as

2 $$ \begin{gathered} F_{11} = \frac{1}{{XX^{\prime } }},\;\;\;{\mkern 1mu} F_{1} = \frac{1}{X} - \frac{1}{{X^{\prime } }},\;\;\;{\mkern 1mu} F_{22} = \frac{1}{{YY^{\prime } }},\;\;\;{\mkern 1mu} F_{2} = \frac{1}{Y} - \frac{1}{{Y^{\prime } }},\;\;\;{\mkern 1mu} F_{33} = \frac{1}{{ZZ^{\prime } }},\;\;\;{\mkern 1mu} F_{3} = \frac{1}{Z} - \frac{1}{{Z^{\prime } }} \hfill \\ F_{44} = \frac{1}{{S_{23}^{2} }},\;\;\;{\mkern 1mu} F_{55} = \frac{1}{{S_{13}^{2} }},\;\;\;{\mkern 1mu} F_{66} = \frac{1}{{S_{12}^{2} }},\;\;\;{\mkern 1mu} F_{12} = - \sqrt {F_{11} F_{22} } ,\;\;\;{\mkern 1mu} F_{13} = - \sqrt {F_{11} F_{33} } ,\;\;\;{\mkern 1mu} F_{23} = - \sqrt {F_{22} F_{33} } , \hfill \\ \end{gathered} $$

where *X* and *X'*, *Y* and *Y'*, *Z* and *Z'* are tensile and compressive strengths of the UD-FRCP along the fiber axial direction (*x*_1_), the transverse direction (*x*_2_), and the thickness direction (*x*_3_), respectively; *S*_12_, *S*_13_, and *S*_23_ are the shear strengths of the UD-FRCP in the *x*_3_, *x*_2_, and *x*_1_ planes, respectively. The related material parameters of the fiber and the matrix are given in Table 1 Ref.^30^.

**Table 1:** Material parameters of fiber and matrix^30^.

Material parameters	*E*_11_ (GPa)	*E*_22/33_ (GPa)	*G*_12/13_ (GPa)	*G*_23_ (GPa)	*X*_T_ (MPa)	*X*_C_ (MPa)	*Y*_T_ (MPa)	*Y*_C_ (MPa)	*SS*_12/23_ (MPa)	*ρ* (g/cm^3^)	ν _12/13_	ν _23_
Fiber	100	93	0.34	0.32	4200	4200	3500	3500	1700	0.97	0.24	0.21
Matrix	3.65	3.65	1.35	1.35	78	146	78	146	156	2	0.35	0.35

The typical deformation and failure behavior of the UD-FRCP after penetration is shown in Fig. 3b, where the full model is re-constructed during post-processing to show the deformation and failure behavior of the UD-FRCP clearly. The residual impact velocities of the projectiles after penetrating the UD-FRCP with various cross-section characteristics can be obtained. Therefore, the dissipated impact energy $\Delta E$, which is used to evaluate the impact protective performance of the UD-FRCP, can be calculated as

3 $$ \Delta E = \frac{1}{2}m\left( {v_{0}^{2} - v_{r}^{2} } \right), $$

where *m*, *v*_0,_ and *v*_r_ are mass, initial velocity, and residual velocity of the projectile, respectively.

For each impact process simulation, it takes nearly 2 h for a computer with an Intel (R) Core (TM) i7-8700 CPU and a memory of 16 GB. As a result, 185 impact processes of the UD-FRCP with various microstructures are simulated and analyzed to build the training set and the testing set to balance the ML accuracy and the computational cost. More cases will be beneficial to improve ML accuracy, but more time-consuming.

### Microstructure characterization

#### Two-point correlation function

**Figure 4:**
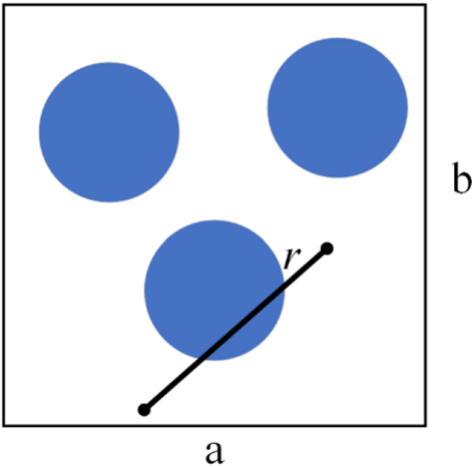
Vector *r* in an arbitrary two-phase microstructure.

It is necessary to extract the specific characteristic data of the cross-sections’ microstructures from the cross-section images of the UD-FRCP, as given in Fig. 2b. Here, the n-point spatial correlation function, which is a measurement of the probability of finding n points in the space occupied by a component within a two-phase material, is employed to analyze the microstructure characteristics of the UD-FRCP. In detail, the one-point correlation function reveals the probability of any point in a type of material; the two-point correlation function reflects the likelihood of two points in a certain distance at the same time; the three-point correlation function indicates the probability of a specific triangle in a particular material. For the present problem, the two-point correlation function is applicable to reveal the microstructure characteristics of the UD-FRCP, such as the fiber content and the relative positions of fiber bundles in the cross-section^31^. The two-point correlation function defines the probability of finding phase *P* and phase *P'* at the head and the tail, respectively, of a vector with a length of *r* that is randomly placed in a microstructure represented by a voxel image with *a* × *b* pixels, as shown in Fig. 4. Each pixel in the image is identified by a unique two-dimensional position vector *s*. Therefore, the two-point correlation function is defined as

4 $$ f_{r}^{{PP^{\prime}}} = \frac{1}{S}\sum\limits_{s = 1}^{S} {m_{s}^{P} } m_{s + r}^{{P^{\prime}}} , $$

where $m_{s}^{P}$ is microstructure function taking the value of either 1 if the material phase *P* is located in the spatial position *s* or 0 if any other different phase is present at the spatial position *s*. *S* denotes the total number of pixels in the image^22^.

In this paper, the inter-fiber spatial correlation is analyzed,

5 $$ f_{r}^{FF} = \frac{1}{S}\sum\limits_{s = 1}^{S} {m_{s}^{F} } m_{s + r}^{F} , $$

where *F* denotes fiber phase.

**Figure 5:**
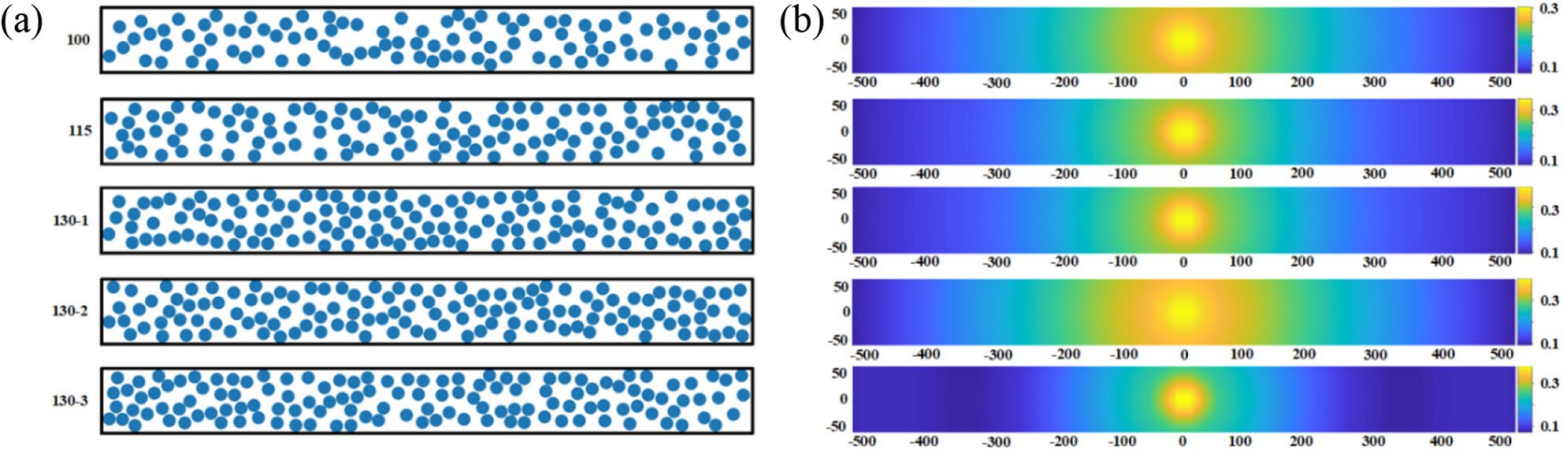
(**a**) Microstructures and (**b**) the corresponding two-point correlation function matrix of microstructures with 100, 115, 130, 130, and 130 fiber bundles corresponding to the fiber contents of 31.41%, 36.11%, 40.84%, 40.84%, and 40.84%, respectively. The labels on the axes refer to the number of pixels.

The microstructure of the composite in the present study is analyzed by 100,000 (1000 × 100) pixels. Figure 5a shows five examples of microstructures with 100, 115, 130, 130, and 130 fiber bundles corresponding to the fiber contents of 31.41%, 36.11%, 40.84%, 40.84%, and 40.84%, respectively. The corresponding two-point correlation matrices (1000 × 100 pixels) of the microstructures are calculated as depicted in Fig. 5b. For the vector *r* with a length of 0 pixel, the value of $f_{r}^{FF}$ represents the content of fibers. The two-point correlation function matrix differs significantly for different fiber contents. Even for the same fiber content as given in Fig. 5, the two-point correlation functions show obvious differences due to the variation of the microstructures. Therefore, the two-point correlation function matrix can describe the characteristics of the composite microstructure. Each microstructure consists of 100,000 values, and a substantial two-correlation function matrix with a dimension of 100,000 × 185 is finally constructed for the total 185 cases.

#### Dimensionality reduction

Since the two-correlation function matrix is massive, it is tough to train the ML model based on the data of the matrix. Therefore, it is essential to reduce the dimensionality but retain the characteristics of the data set. Here, the principal component analysis (PCA)^32^, which is helpful for extracting the main features and reducing the size of the data set, is taken to shrink the dimensionality of the data set of the two-correlation function matrix. The principal components of a data set are found through linearly transform from a series of possible related variables to some unrelated variables. To ensure that the first principal component describes the direction of maximum variance, the data set has been centralized. According to the zero empirical mean theory^33^, the first weight vector **w**_1_ of the data set **X** can be defined as

6 $$ {\mathbf{w}}_{1} = \mathop {\arg \max }\limits_{\left\| w \right\| = 1} {\text{Var}}\left\{ {{\mathbf{w}}^{{ \top }} {\mathbf{X}}} \right\} = \mathop {\arg \max }\limits_{\left\| w \right\| = 1} E\left\{ {\left( {{\mathbf{w}}^{{ \top }} {\mathbf{X}}} \right)^{2} } \right\} $$

which represents the maximum variance of ${\mathbf{w}}^{{ \top }} {\text{X}}$ when taking **w** = **w**_1_.

For finding the *k*th principal component, where *k* ≥ 2, the previously obtained principal components need to be removed from the data set **X**, and this process can be depicted as follow,

7 $$ \begin{gathered} \widehat{{\mathbf{X}}}_{k - 1} = {\mathbf{X}} - \sum\limits_{i = 1}^{k - 1} {{\mathbf{w}}_{i} } {\mathbf{w}}_{i}^{{ \top }} {\mathbf{X}} \hfill \\ {\mathbf{w}}_{k} = \mathop {\arg \max }\limits_{{\left\| {\mathbf{w}} \right\| = 1}} E\left\{ {\left( {{\mathbf{w}}^{{ \top }} \widehat{{\mathbf{X}}}_{k - 1} } \right)^{2} } \right\} \hfill \\ \end{gathered} $$

The minimum *k* is determined according to the maximum mean–variance of the data set,

8 $$ \frac{{\frac{1}{m}\sum\limits_{i = 1}^{m} {\left\| {x^{(i)} - x_{{{\text{approx}}}}^{(i)} } \right\|^{2} } }}{{\frac{1}{m}\sum\limits_{i = 1}^{m} {\left\| {x^{(i)} } \right\|^{2} } }} \le e, $$

where *m* is the total data of the data set, $x^{(i)}$ and $x_{{{\text{approx}}}}^{(i)}$ are the *i*th actual value and estimated value. *e* can be chosen by specific demand according to the trade-off between dimension and precision. The smaller the dimension is, the less accuracy in model training is achieved. In the present study, *e* is taken as 0.01 to ensure adequate accuracy for the main characteristics of the microstructures, and therefore *k* is determined as 10.

### Machine learning algorithms

#### Gradient boosting regression algorithm

The gradient boosting regression (GBR) algorithm combines “weak learners” into a “strong learner” in an iterative manner^34^. At each stage of gradient boosting *m*, 1 ≤ *m* ≤ *M*, assuming there is already an imperfect model *F*_*m*_, the next-step model *F*_*m*+1_ of the GBR algorithm is improved by adding a new estimator *h*^35^,

9 $$ F_{{m{ + }1}} \left( x \right) = F_{m} \left( x \right) + h\left( x \right), $$

For finding *h*, gradient boosting is based on the following observation that a perfect *h* can ultimately diminish the residual error of the current imperfect model *F*_*m*_,

10 $$ F_{m + 1} \left( x \right) = F_{m} \left( x \right) + h\left( x \right) = y, $$

where *y* is the real value of the problem.

Therefore, gradient lifting obtains *h* by fitting *y*-*F*_*m*_(*x*). Like other variants of lifting methods, *F*_*m*+1_ becomes more accurate by correcting the error of *F*_*m*_. The idea is originated from the gradient descent method. At first, newly added weak classifiers are trained based on the negative gradient information of the current model loss function, and then combines the trained weak classifiers into the existing model in an additive form. In short, the prediction model produced by GBR is an integration of weak prediction models.

#### Support vector regression algorithm

Support vector regression (SVR) algorithm constructs hyperplanes or hyperplane sets in high or infinite-dimensional spaces, which can be used for classification regression or other tasks^36^. Intuitively, the farther the classification boundary is from the nearest training data point, the better the model because it can reduce the generalization error of the classifier. The SVR is trying to find a hyperplane that minimizes the distance from all data to the hyperplane.

#### Random forest regression algorithm

The random forest regression (RFR) is a classifier that contains multiple decision trees, and its output category is determined by the mode of the sum of all the individual trees^37^. RFR randomly uses variables and data of a data set to generate many classification trees that form a forest, and the prediction result is obtained by weighting the individual classification tree.

## Results and discussion

### Construction of data set

**Figure 6:**
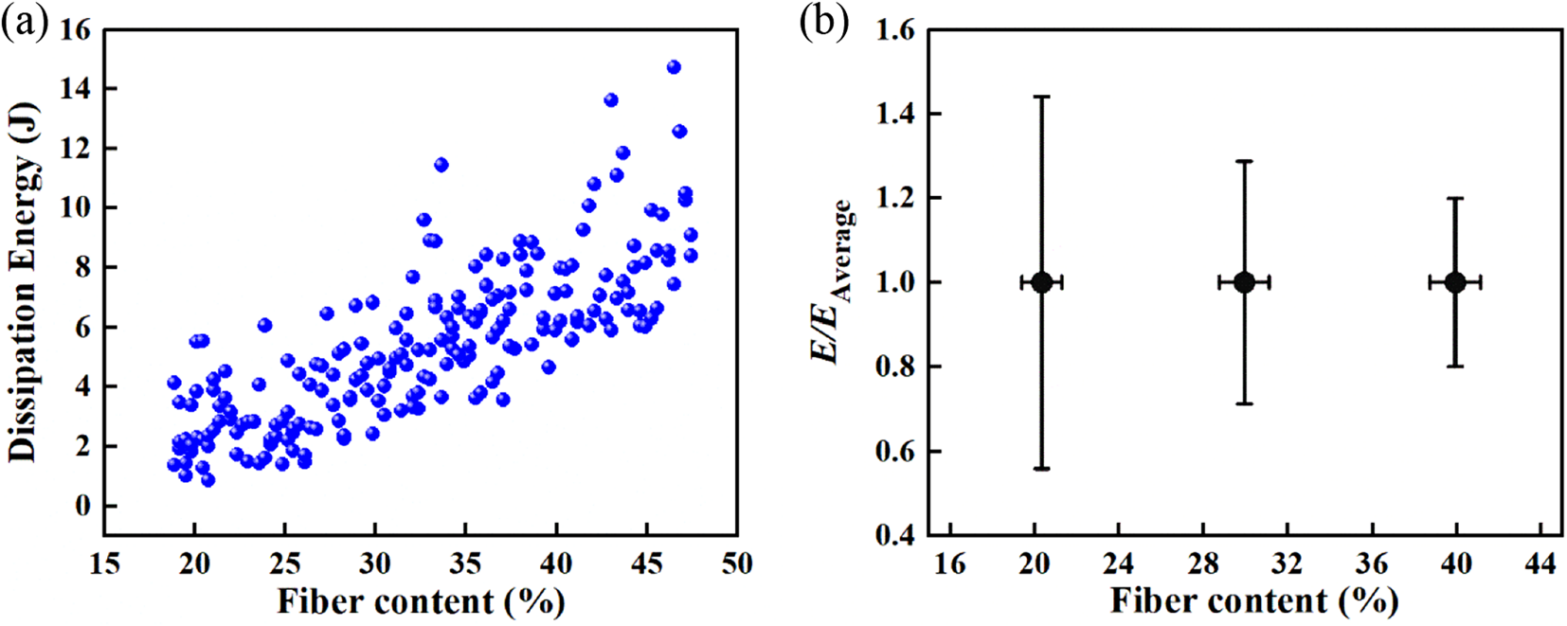
Anti-penetration performance of the micro-scale model. (**a**) The relationship between the dissipation of impact energy and fiber content. (**b**) The relationship between normalized dissipation of energy *E/E*_Average_ and fiber content.

As shown in Fig. 6a, the dissipated energy of the UD-FRCP for various fiber contents are summarized based on the 185 impact simulation cases. It can be seen that the impact energy dissipation of the UD-FRCP increases from 2.83 to 7.14 J with increasing the fiber content from 20.34 to 39.92%, which indicates that the properties of UD-FRCP are rising with the more fiber content. The dissipated energy for the fiber content of 42% is almost three times larger than that for the fiber content of about 24%. In addition, although the total dissipated energy of the UD-FRCP is dispersed, we can get the tendency from the result. The dispersion of normalized dissipated energy, obtained by using FEA results divide the corresponding interval average value, is diminishing with increasing the fiber content as shown in Fig. 6b, where the normalized dissipated energy for the fiber contents in the range of 18 ~ 22%, 28 ~ 32%, and 38 ~ 42% are given. For low fiber contents of 18 ~ 22%, the data divergence can be as high as 45%, while it decreases to 20% for relatively high fiber contents of 38 ~ 42%. The divergence of dissipated impact energy of the UD-FRCP for the same fiber content should be ascribed to the difference of two-point function values of microstructures introduced by the uncertainty of fiber bundle positions in the cross-section. For low fiber contents, high uncertainty of fiber bundle positions leads to a noticeable difference of two-point correlation function values and finally results in an increased divergence of dissipated impact energy. Therefore, besides the fiber content, it is vital to consider the effects of microstructures on the impact protective performance of composites.

### The sensitivity of parameters of the training algorithms

The model is trained by the GBR, SVR, and RFR algorithms in Scikit-learn^38^ to study the difference of training algorithms and determine the appropriate algorithm for the present problem. We randomly choose 175 cases as a training data set during training the model and the rest 10 cases as the testing set. According to the definition of the No Free Lunch Theorem, it is known that no algorithm and parameter is suitable for all data sets^39^. Therefore, it is crucial to obtain the optimized parameters for a given algorithm to predict better. Consequently, the sensitivity analysis of the critical parameters for GBR, SVR, and RFR algorithms are performed.

For the GBR algorithm, the fundamental parameters include the learning rate, the number of estimators, and the maximum depth of a single decision tree. In the present study, the learning rate is determined as 0.001 to ensure that the optimal parameters can be obtained while minimizing the computation cost. We mainly focus on the effects of the number of estimators and the maximum depth of a single decision tree on the training results of the GBR algorithm. For the RFR and the SVR algorithms, the sensitivities of the number of estimators and the kernel functions are investigated, respectively.

**Figure 7:**
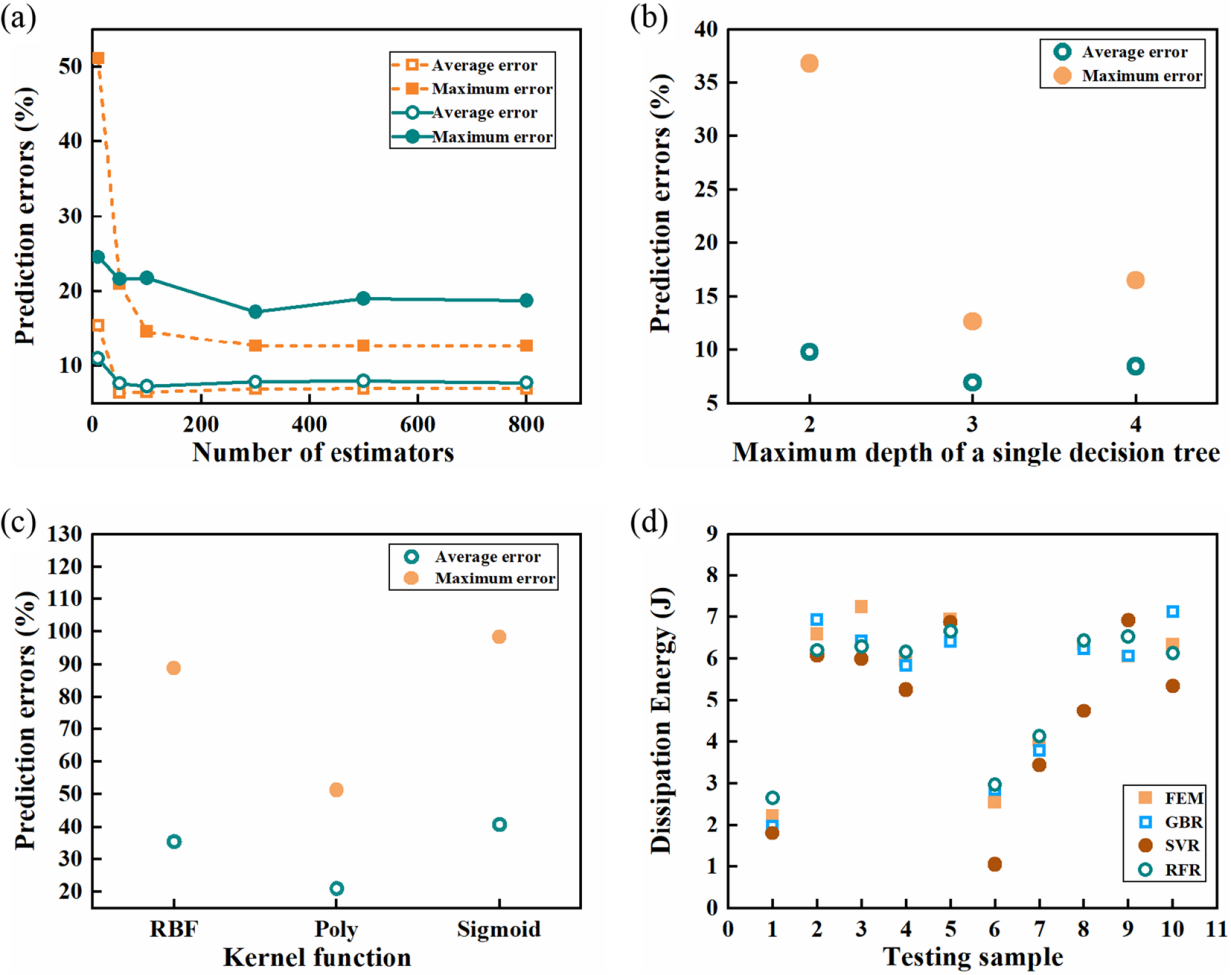
FEM results and prediction results.

The sensitivities of the number of estimators and the maximum depth of a single decision tree on the training results of the GBR algorithm are shown in Fig. 7a,b, respectively. From Fig. 7a, it can be seen that the average error decreases quickly from 15.47 to 6.94%, and the maximum error decreases abruptly from 51.16 to 12.69% with increasing the number of estimators from 10 to 300. After that, the average and the maximum errors approach the plateaus at 6.94 and 12.69% as the number of estimators continues to increase. Generally, the number of estimators is equivalent to how many weak classifiers are used in the model. When the number of classifiers reaches a critical value, the accuracy of the training model will be saturated, resulting in the plateaus of errors with the continual increase of the number of estimators, as observed in Fig. 7a. Therefore, the number of estimators is determined as 300 for the GBR algorithm. As shown in Fig. 7b, the effect of the maximum depth of a single decision is non-monotonic. The average error decreases from 9.76 to 6.94%, and the maximum error decreases rapidly from 36.82 to 12.69% with increasing the maximum depth of a single decision tree from 2 to 3, then both the average and the maximum errors increase slightly with continually increasing the maximum depth of a single decision tree to 4. It can be well understood by the fitting ability of the GBR with increasing the maximum depth of a single decision tree. When the depth of a single decision tree is too small, the characteristics of the data set cannot be fitted well, whereas if it is too large, excessive parameters are introduced in the GBR algorithm, leading to unsatisfied fitting results due to the limited training data set, which we call over-fit^40^. As a result, the appropriate depth of the single decision tree is determined as 3 for the GBR algorithm in the present study.

The effect of the number of estimators on the training results of the RFR algorithm is also depicted in Fig. 7a. Similar to the GBR algorithm, the average and the maximum errors for the RFR algorithm decrease with increasing the number of estimators, and then they gradually decay to stable values at 7.87 and 17.26%, respectively, for the number of estimators more than 300. Therefore, the number of estimators is also determined as 300 for the RFR algorithm. The influences of various kernel functions that are radial basis function (RBF), polynomial function (Poly), and sigmoid functions (Sigmoid), on the training results of the SVR algorithm, are shown in Fig. 7c. The average and the maximum errors are 35.42 and 88.81% for RBF, 21.07 and 51.34% for Poly, 40.85, and 98.32% for Sigmoid, respectively. It can be seen that the training results with Poly are superior to those with the RBF and Sigmoid for the SVR algorithm, and therefore the Poly is used for the prediction by the SVR algorithm.

The prediction results from the GBR, the RFR, and the SVR algorithms are given in Fig. 7d, and the average and the maximum errors are listed in Table 2. It is clearly shown that the GBR algorithm is more appropriate for the present study in terms of the average and the maximum errors when compared to the RFR and the SVR algorithms. The GBR algorithm gives the best predictions with an average error of 6.94% and a maximum error of 12.69%, as listed in Table 3, followed by the RFR algorithms with an average error of 7.87% and a maximum error of 19.61%. The SVR algorithm arises the worst predictions with an average error of 18.49% and a maximum error of 58.77%, which is several times larger than the results of the GBR algorithm and unacceptable for the present study. Such a significant error of the SVR algorithm should be ascribed to the intrinsic incapability of support vector algorithms as the SVR algorithm for the non-continuous regression problems, as in the present study. For a support vector algorithm, a data set is classified on a multi-dimensional plane to find the hyperplane and the maximum distance. If it is applied to a non-continuous regression problem, it is difficult to find the hyperplane, and the results of each category are voted to finally obtain an average value, leading to a relatively large error. In contrast, the GBR and the RFR algorithms in the Scikit-learn package are based on decision trees, which are suitable for dealing with non-continuous regression problems as employed in the present study. Specifically, for the RFR algorithm, the data set is randomly sampled into the decision trees, and the prediction result is averaged by all the votes of the multiple decision trees. While for the GBR algorithm, a first randomly selected decision tree is used to fit the data set, and the training error is then substituted into the second chosen decision tree randomly, and so on. With this method, the training result finally approaches the actual value step by step. As a result, both the GBR and the RFR algorithms can give good predictions, and the GBR algorithm is more appropriate for predicting the impact of the proactive performance of UD-FRCP. Further ML study will be performed to predict the dynamic mechanical behavior of other types of composites, such as two- or three-dimensional fabric composites, in the future.

**Table 2:** Errors of various training algorithms.

Algorithm	GBR	SVR	RFR
Average error (%)	6.94	18.49	7.87
Maximum error (%)	12.69	58.77	19.61

**Table 3:** Prediction results of the ML model with the GBR algorithm.

Sample	1	2	3	4	5	6	7	8	9	10
Error (%)	11.20	5.14	11.14	1.85	7.58	12.39	5.77	1.73	0.32	12.69

### The effect of the number of cases of the GBR algorithm

In addition, we explored the effect of the number of training cases on the prediction error. We chose the training cases and predicting cases randomly, and ensured the number ratio of predicting cases to training cases almost constant for comparison. It can be seen that the average and maximum errors basically decrease as the training cases increases as given in Table 4, and an acceptable accuracy is reached for 175 training cases and 10 predicting cases.

**Table 4:** Effect of the number of training cases.

Training cases	100	120	140	160	175
Predicting cases	6	7	8	9	10
Average error (%)	16.91	16.18	14.13	12.63	6.94
Maximum error (%)	60.60	51.10	51.94	36.36	12.69

### The discussion on the uncertainty of the prediction

The uncertainties of the data set can be categorized into two aspects: aleatoric uncertainty and epistemic uncertainty when constructing the prediction model by using a finite size training set^41^. Aleatoric uncertainty is determined by the quality of the data set and the essence of the prediction task. There are inevitable measurement errors resulted from the precision of instruments while collecting data. Epistemic uncertainty is coming from the training process for the reason that it has intrinsic error between the prediction value and the true value due to the limitation of data size. The error will decrease with increasing the amount of training data.

In this work, the relationship between the microstructure and the penetration resistance of UD-FRCP was investigated. The data set was built by extracting the characteristics of the microstructure as input data. The absorbed energy of the composite after perforation were used in the FEA as output data. The error caused by anthropogenic factors is non-existent because the same simulation conditions are used for all of the cases. Therefore, it is relatively reliable in terms of aleatoric uncertainty. For the epistemic uncertainty in this work, with the increase of training cases, the training data with random 175 cases are close to the distribution of the task, and the machine learning model can predict a value close to the true value with a maximum error of 12.69%. Therefore, it is also acceptable in terms of epistemic uncertainty. In summary, the machine learning model in this paper can effectively predict the penetration resistance of UD-FRCP by microstructure recognition.

## Conclusions

The ML method is employed to predict the impact protective performance of UD-FRCP for the first time. The main conclusions can be summarized as follows.

1. A ML model is developed based on the 185 micro-scale simulation results to predict the macroscopic impact protective performance of UD-FRCP. The average error is 6.94%, and the maximum error is 12.69%, which is acceptable for ballistic impact problems of composites, providing a high-efficient method for analyzing the dynamic behavior of UD-FRCP.
2. The sensitivities of the critical parameters of training algorithms are investigated. The results showed that the increase in the number of estimators for GBR and RFR could increase the accuracy of the model, and the critical number of 300 is observed for the saturation of the accuracy. The increase of the maximum depth of a single decision tree for the GBR algorithm shows non-linear effects on the accuracy of the model.
3. The decision trees based algorithms are appropriate for the present study problem, and the GBR algorithm is found to give the best prediction results. More ML models will be built for other types of composites in the future.
4. The number of 185 cases is appropriate for this problem compared to other numbers of cases. The accuracy is guaranteed without a very large computational effort.

The relationship between microstructures and the impact protective performance of UD-FRCP is built in this paper by taking the GBR algorithm in the ML model, which can predict the impact resistance and energy dissipation ability of UD-FRCP accurately. For a large diversity of microstructures in design, this ML method as put forward in this paper can ensure efficiency and accuracy. The other factors such as type of fibers, different matrix materials, and even various micro-structural topologies can be considered in the future. It provides a good solution for the rapid design of composite with high protective performance.

## Data Availability

The data that support the findings of this study are available from the corresponding author upon request.
